# Dual-layer spectral CT fusion imaging for lung biopsies: more accurate targets, diagnostic samplings, and biomarker information?

**DOI:** 10.1186/s41747-022-00290-0

**Published:** 2022-08-15

**Authors:** Marco Curti, Federico Fontana, Filippo Piacentino, Christian Ossola, Andrea Coppola, Giulio Carcano, Massimo Venturini

**Affiliations:** 1 Diagnostic and Interventional Radiology, Circolo Hospital and Macchi Foundation, Insubria University, Varese, Italy; 2 Department of General, Emergency and Transplants Surgery, Circolo Hospital and Macchi Foundation, Insubria University, Varese, Italy

**Keywords:** Decision making, Biomarkers, Biopsy (needle), Lung neoplasms, Tomography (x-ray computed)

## Abstract

The increasingly widespread use of computed tomography (CT) has increased the number of detected lung lesions, which are then subjected to needle biopsy to obtain histopathological diagnosis. Obtaining high-quality biopsy specimens is fundamental for diagnosis and biomolecular characterisation that guide therapy decision-making. In order to obtain samples with high diagnostic potential, fusion imaging techniques, such as fusion between positron emission tomography and CT, have been introduced to target the biopsy where there more viable neoplastic cells can be sampled. Nowadays, dual-layer spectral CT represents a novel technology enabling an increased tissue characterisation. In particular, *Z*-effective images, *i.e.*, colour-coded images based on the effective atomic number of tissue components, provide a higher level of discrimination than usual imaged based on x-ray attenuation in Hounsfield units and offer the potential of a better tissue characterisation. Our hypothesis is based on the future use of data provided by spectral CT, in particular by *Z-*effective images, as a guide for appropriate biopsy sampling for histopathological and biomolecular characterisation in the era of patient tailored-therapy.

## Key points

- Spectral computed tomography (CT) is an innovative diagnostic tool.
- CT-guided lung needle biopsy is a key procedure in the diagnostic and therapeutic pathway.
- Dual-layer spectral CT fusion imaging could improve the quality of the biopsy sample.

## Background

In 2020, lung cancer was the second most common tumour and the leading cause of cancer-related deaths in the USA [1]. Mortality remains very high despite the many therapeutic approaches available such as surgery [2], radiotherapy [3], ablation [4], chemotherapy [5], and immunotherapy [6], alone or variously combined with each other. Screening programs have been largely developed worldwide to early diagnose lung cancer and prevent its metastatic spreading [1, 7]. Moreover, in recent years, the increasing use of computed tomography (CT) led to a rise in detection of pulmonary nodules and lung malignancies diagnosis [8]. An accurate evaluation of the patient’s risk factors and the CT characteristics of the nodule is usually performed but biopsy still remains the standard of care for a certain diagnosis and a subsequent treatment choice [9].

In 2019, The Lung Imaging Reporting and Data System Lung-RADS [10] was updated from 1.0 to 1.1 version, allowing a better standardisation of lung CT reporting and indications for further diagnostic examination or tissue sampling. In the era of targeted therapies, the role of percutaneous biopsy has gained even more a central role beside the initial histopathological diagnosis, especially considering the role of specific biomarkers guiding therapeutic decisions for many solid tumours [11]. This aspect implies a more extensive involvement of radiologists in the context of multidisciplinary discussions and in the planning of clinical-therapeutic pathways.

Percutaneous lung needle biopsy can be guided by different imaging techniques, such as CT [2], fluoroscopy CT [12], cone beam CT [13], positron emission tomography (PET)-CT [14], or ultrasound in case of superficially located lesions [15]. The choice of the imaging technique depends on its availability, lesion type and location, patient’s collaboration, and operator’s preferences [2]. Lung needle biopsy is a well-established procedure [16], but it may be not diagnostic for several reasons: low percentage of tumour cells, high percentage of necrosis or non-target sampling. The diagnostic accuracy is obviously poorer in case of subcentimeter nodules [17].

Nowadays, sophisticated software allowing fusion imaging with different methods (CT, magnetic resonance and PET-CT) have improved diagnostic accuracy [8, 13]. The capability to fuse morphological information obtained with classical radiological methods and metabolic activity provided by nuclear medicine techniques such as PET seems to be advantageous. In particular, the fusion between fluoro-CT (or cone beam CT) and PET-CT images has proven to be very useful in guiding biopsy procedures, increasing their diagnostic accuracy. Indeed, the intensity of accumulation of the tracer correlates with biological activity, facilitating the identification of the most active tumour regions [8, 13]. PET is an effective, reliable, and highly diagnostic method, but it is still quite expensive and not a widely available imaging method. A prolonged waiting time can be dangerous in fragile patients such as cancer patients, as it may represent a diagnostic delay.

### Hypothesis

A new, latest-generation spectral CT system with dual-layer detector was recently installed at our centre (IQon, Philips Healthcare, Best, the Netherlands. This equipment allows to obtain the spectral separation at the level of a dual-layer detector [18], providing images based on iodine density, virtual non-contrast, and effective atomic number (*Z*) [18]. These applications permit to enhance vascular contrast, to reduce image artifacts, and to evaluate composition of different tissues or lesions using iodine map and *Z-*effective images [18, 19]. In addition, these applications allow both a possible reduction in radiation dose and amount of contrast agent to be administered [19, 20]. *Z-*effective images are colour-coded images based on the effective atomic number of the tissue; this information is obtained from the spectral decomposition process into photoelectric and Compton scattering data [19]. The atomic number of a tissue is also correlated by the presence of iodine within it [18].

**Fig. 1:**
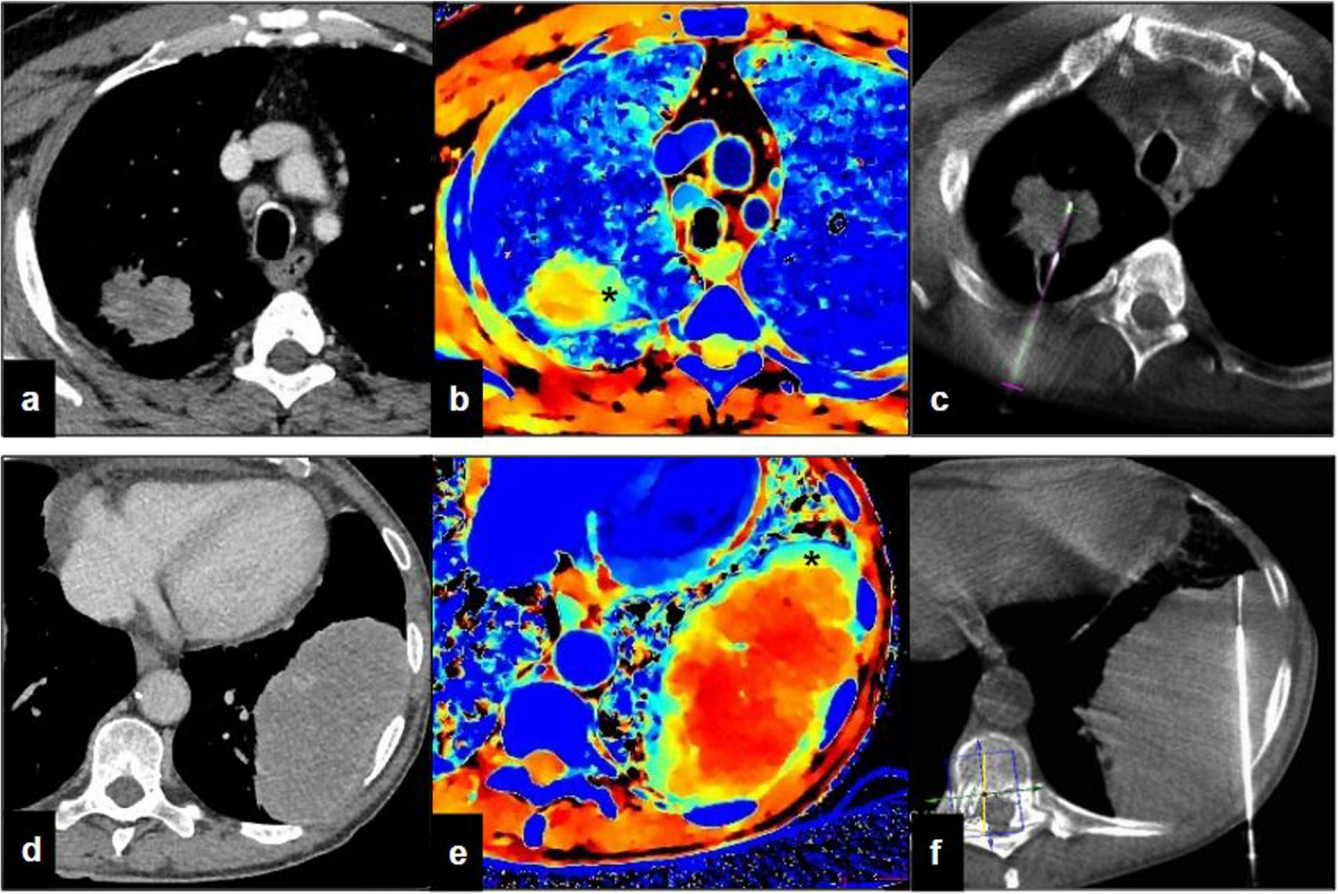
Percutaneous lung biopsy. **a** Axial routine diagnostic image in a patient with solid lung lesion at the upper right lobe. **b** Effective atomic number-based reconstruction at the same level: the lesion is characterised by inhomogeneous intralesional atomic number with a central area showing a reduced atomic number (yellow–red) and a medial eccentric area showing a higher atomic number (light blue, asterisk). **c** Intraprocedural cone beam computed tomography (CBCT) shows that the needle has been located in the area with the highest atomic number. **d** Axial routine diagnostic image in a patient with solid lung lesion at the lower left lobe. **e** Effective atomic number-based reconstruction at the same level: the lesion is characterised by inhomogeneous intralesional atomic number with a central area showing a reduced atomic number (yellow–red) and an external circular area showing a higher atomic number (light blue, asterisk). **f** Intraprocedural CBCT shows that the needle has been located in the area with the highest atomic number

In our preliminary experience based on a small cohort of patients with lung lesions greater than 3 cm, we have observed an inhomogeneous *Z-*effective value within the lesion (Fig. 1). To our knowledge, similar data have been never reported. Highly vascularised areas are those with higher *Z* values after iodinated contrast medium injection. We hypothesise to use *Z*-effective images as a guide to biopsy large lesions with an inhomogeneous atomic number assuming that poorly vascularised areas are potentially less diagnostic due to necrosis and fibrosis then hypervascular areas. Therefore, future applications would be to fuse *Z*-effective data with cone beam CT images during the biopsy procedure, to combine the functional information obtained with spectral CT and the biopsy guidance, increasing the diagnostic power of the biopsy.

Summarising, the dual-layer spectral CT system using the *Z-*effective images as lung biopsy guide might be helpful to provide more diagnostic samplings and biomarkers information in the era of patient-tailored therapy [21, 22]. Obviously, clinical data will be necessary to support this hypothesis.

## Data Availability

Not applicable.

## Abbreviations

CT: Computed tomography
PET: Positron emission tomography
